# The role of medical schools in promoting social accountability through shared decision-making

**DOI:** 10.1186/2045-4015-3-26

**Published:** 2014-07-22

**Authors:** Orit Karnieli-Miller, Yaara Zisman-Ilani, Dafna Meitar, Yoseph Mekori

**Affiliations:** 1 Department of Medical Education, Sackler School of Medicine, Tel-Aviv University, Tel-Aviv, Israel; 2 Department of Community Mental Health, Faculty of Social Welfare and Health Sciences, University of Haifa, Haifa, Israel; 3 Dean, Sackler Faculty of Medicine, Tel-Aviv University, Tel-Aviv, Israel

## Abstract

Reducing health inequalities and enhancing the social accountability of medical students and physicians is a challenge acknowledged by medical educators and professionals. It is usually perceived as a macro-level, community type intervention. This commentary suggests a different approach, an interpersonal way to decrease inequality and asymmetry in power relations to improve medical decisions and care. Shared decision-making practices are suggested as a model that requires building partnership, bi-directional sharing of information, empowering patients and enhancing tailored health care decisions. To increase the implementation of shared decision-making practices in Israel, an official policy needs to be established to encourage the investment of resources towards helping educators, researchers, and practitioners translate and integrate it into daily practice. Special efforts should be invested in medical education initiatives to train medical students and residents in SDM practices.

## 

In their 2014 paper, Rudolf et al. [1] provided medical leaders, educators and policymakers an opportunity to reflect upon the responsibility and accountability of medical schools toward the community. As they concluded, medical schools in Israel are focused on what Boelen et al. [2] defines as "social responsibility" and "responsiveness". In fact, medical education initiatives in Israel have been limited only to providing knowledge about health inequalities and social determents of health, as well as specific actions focused on local community needs [1]. According to Rudolf’s analysis, Israeli medical schools are not educating students about what Boelen considers “true social accountability,” which involves working in partnership with the communities about their perceived health needs and accordingly defining the medical school’s academic agenda. To change this situation, they suggest that medical schools need to focus on educating their students to recognize the physician’s role in tackling health inequities. Following their suggestion, this commentary focuses on an interpersonal approach to achieving true social accountability and the unique role of medical schools in leading these efforts. We would like to suggest Shared Decision-Making (SDM) practices as a model that provides an opportunity to partner with a patient, collaboratively identify his or her needs, and then use this shared understanding to directly improve their care. We believe that partnership on the individual level will positively influence and impact the general community [3,4].

SDM is a central component of patient-centered care [5-7]. It engages both physician and patient in an interactive process in which information is shared, discussion and deliberation is encouraged and a shared agreement regarding the treatment plan is reached [8,9]. The resulting decision integrates patients’ medical needs with their values, preferences, life-plan and goals [8-11]. Beyond merely sharing information or providing medical knowledge from one party to the other, SDM is about enhancing patients and physicians' mutual engagement in the decision-making process while remaining attuned to the patient’s personhood. In this short piece we will not be able to provide an in-depth exploration of SDM practices and complexities. Instead, we will focus on the potential of SDM to minimize power disparities, equalize the voices of patients and physicians, and ultimately improve care for otherwise disadvantaged populations, furthermore we will discuss the important role of medical schools in integrating the teaching of SDM values and practices.

At the heart of the SDM model lies the "partnership principle". This notion describes the formation of a strong relationship, a partnership, between a patient and his or her physician that enhances trust and improves adherence to the subsequent treatment plan [12,13]. Partnerships are particularly important in medical encounters, as the relationship formed directly influences the direction of information flow between the parties [13]. Each party possesses a distinctly important type of information: the physician has knowledge about the disease, its related symptoms, possible implications, treatments and their side effects, while the patient has knowledge regarding her/his own values, perceptions, barriers, preferences and personal experience living with the disease [9,14,15]. When a partnership is achieved on an interpersonal level between patient and physician, information flow becomes bidirectional, and these two crucial levels of information can be shared. Therefore, partnership in the SDM model represents a shift from the hierarchical asymmetrical one-way communication toward a more equal two-way communication [8,9].

The reduced informational asymmetry enabled by SDM is important for all populations, but especially for disadvantaged ones where this asymmetry is even more apparent [16,17]. Several studies have found a connection between patients' socioeconomic status (SES) and the corresponding communication style used by physicians. Patients with a low SES received less information from physicians about their disease and treatment options than patients with higher SES, and were less likely to be invited to participate in the decision-making process [18,19]. Thus, professionals tend not to implement SDM practices, and specifically not to share information, with low SES patients. One promising strategy to reduce such asymmetry in information sharing and increase patient involvement in decision-making is to train medical students and residents to recognize these tendencies and consciously deliver information to all patients in an understandable manner [20]. This requires training in communication skills that include tailoring information to the patient's health literacy level and specific cultural needs, providing balanced information about risks and benefits, and eliciting patient preferences. The development of these critical skills by medical students and professionals will provide future patients with more opportunities to be involved in their own care, and hopefully take responsibility for their own health.

One of the difficulties that underserved populations experience is disempowerment. In the context of healthcare, empowerment occurs by sharing information that will enhance the confidence and understanding of the patient, and by providing an arena in which the patient can share personal information that is relevant to the illness or treatment plan. According to Rowlands [21], “empowerment is more than participation in decision-making; it is more about the processes that provide people with the understanding that they are able and entitled to make decisions” (p. 14). Empowerment is enhanced by the physician's willingness to listen to the patient, their ability to elicit, identify and acknowledge the patients perspective, situation and psychosocial issues and barriers, as well as their ability and willingness to provide the patient with needed information about the relevant diagnoses and treatment options upon their request [22]. When this process is carried out in a respectful manner, patients are empowered by being involved in their treatment and related decisions.

In the Israeli context, SDM practice lags behind other western countries [23]. The teaching and practice of SDM in Israel is rare [23,24] and only one randomized control study of SDM has been recently completed [Zisman-Ilani, Roe, & Karnieli-Miller: The effectiveness of a new shared decision-making intervention for psychiatric hospitals, in preparation]. This is mainly because there is no official policy that enables adequate implementation of SDM, which translates to limited resources to apply and assess SDM as a viable healthcare model.

To increase the use of SDM practices in Israel, various related efforts need to be focused on helping educators, researchers and practitioners (see Table 1). These include training in basic communication skills and specifically for SDM practices; researching and assessing the application of SDM; and developing SDM interventions, decisional aids, and other sources of data visualization that can help patients acquire the necessary information in a friendly simple manner to become involved in the medical process [22,23,25,26].

**Table 1:** Actions to promote implementation and validation of Shared Decision Making (SDM)

**Areas of focus**	**Proposed interventions**
**Education**	Establish a training protocol on how to teach SDM values and practices
	Integrate SDM competency training into pre-clinical and clinical medical school curriculum
	Enhance SDM competencies in residency training and continuing medical education in general
**Research**	Validate and develop Hebrew assessment tools for SDM that can help evaluate and guide practice
	Explore and identify different patients' needs and preferences regarding involvement in medical decision-making
	Develop, disseminate and assess interventions to facilitate SDM in routine practice
**Practice**	Disseminate formal training and practical tools to allow the application of SDM practices in routine care (including: how to create partnerships, how to communicate risks and benefits of different treatment options, how to explore patients' values, needs, preferences and barriers)
	Design and distribute decisional aids that can provide patients with medical information about treatment options, benefits, and side effects in lay language

Due to the important role SDM may have, and the unique and important skills it requires, training of SDM practices should be incorporated early on in medical schools. Through early integration, in undergraduate educational curriculums, medical graduates may establish these skills as part of their interaction practices and their communication habits [27-29]. However, this training is not common in medical schools [29,30].

Several medical schools, in Israel and around the world, have begun to take steps in this direction. For example, at Tel Aviv University, through various initiatives of the Department of Medical Education, we have started to teach various components of SDM. We teach its theoretical principals and components during the first year; we guide students to practice SDM in identifying specific personal goals with patients they meet throughout the second year in their "therapeutic relationship" project. During this project each student meets a patient, usually from an underserved population, and is requested to collaboratively identify a need and define a goal that requires an intervention aimed at improving the quality of life of the patient. This process includes shared understanding of the strengths, barriers and health related needs and shared agreement about the intervention. In our communication skills courses, throughout the years, we provide training and feedback about building partnerships, eliciting patients’ preferences and values and sharing balanced information respectfully and clearly. We hope to enhance SDM practices through these direct experiences; however, we feel that there is still much more to do.

Furthermore, we believe that it is time that the various medical schools in Israel will share ideas and practices used to teach SDM. Collaborating to identify and develop the best methods to teach and integrate SDM into the curriculum. If all our students from all medical schools will be equipped with the skills to practice SDM they will later on be able to apply it in the hospitals and the community.

To summarize, SDM is an interpersonal model that can potentially help patients and physicians (and other health professionals) achieve more than just the best treatment decision. This is especially true for disadvantaged populations who lack educational or financial means to attain better care. It enables a unique collaboration, a partnership that reduces asymmetry in medical encounters, empowers patients, and provides them with a direct opportunity to be involved in their care. Integrating SDM practices early on in medical schools and in residency programs can enhance the chances of acquiring these skills and implementing them later on in practice. If SDM will be practiced correctly, this model can help practitioners tailor their care according to individual patients' health needs as well as encourage all patients to take responsibility and be accountable for their own health. By enhancing the patient-physician partnership on the interpersonal level, the overall health of the community can be improved.

## Competing interest

All authors declare that they have no competing interests.

## Authors' information

*Dr. Orit Karnieli-Miller* is a PhD, senior lecturer and a social worker in the Department of Medical Education, at Sackler School of Medicine. She specializes in research focusing on communication in healthcare. Her research is on the complex communication challenges of diagnostic disclosure, handling multi-participant conversations, shared decision-making in medical practice, and professionalism in medical school training.

*Yaara Zisman-Ilani* is a Ph.D. student and a psychologist interested in patient centered-care. In her Ph.D. study she developed a shared decision-making intervention and implemented it in psychiatric hospitals.

*Dr. Dafna Meitar,* is an MD, a senior teacher and coordinator in the Department of Medical Education. She is a Children's Palliative Care physician and an expert in teaching communication skills.

*Prof. Yoseph Mekori* serves as the Dean of the Sackler Faculty of Medicine and Head, Department of Internal Medicine, Meir Hospital, Kfar Saba.

## Commentary on

Rudolf MC, Reis S, Gibbs TJ, Eaton DM, Stone D, Grady M, et al. How can medical schools contribute to bringing about health equity? Israel Journal of Health Policy Research. 2014;3:17.
